# Aromatic Anion Carrier via Self-Assembly with Imidazolium-Fused Aromatic Amphiphiles

**DOI:** 10.1021/prechem.4c00074

**Published:** 2024-12-18

**Authors:** Jung Yeon Park, Dongjun Baek, Hyunggeun Min, Bongjun Yeom, Jeong Sook Ha, Yongju Kim

**Affiliations:** † KU-KIST Graduate School of Converging Science and Technology, Korea University, Seoul 02841, Republic of Korea; ‡ Department of Chemical Engineering, Hanyang University, Seoul 24763, Republic of Korea; § Department of Chemical and Biological Engineering, Korea University, Seoul 02841, Republic of Korea; ∥ Department of Integrative Energy Engineering, Korea University, Seoul 02841, Republic of Korea; ⊥ Chemical and Biological Integrative Research Center, Korea Institute of Science and Technology, Seoul 02792, Republic of Korea

**Keywords:** vesicle, aromatic anion carrier, aromatic amphiphiles, imidazolium, cellular uptake, fluorescent supramolecular materials

## Abstract

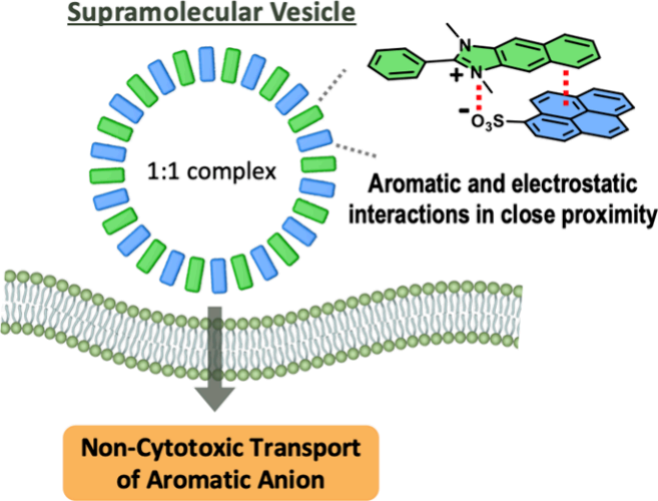

The transport of anions across cell membranes is difficult because of the negatively charged outer surfaces of cell membranes. To overcome this limitation, herein, we report a system for transporting aromatic anions across cellular membranes via self-assembly using a synthetic imidazolium-fused aromatic amphiphile. The amphiphile with cationic and aromatic groups in close proximity to each other could interact with anionic pyranine via electrostatic and aromatic interactions to form supramolecular vesicles. Supramolecular vesicles based on the synthetic imidazolium-fused aromatic amphiphile and pyranine complex transport anionic aromatic pyranine across the membranes of live MCF-7 cells without cytotoxicity.

## Introduction

Cell membranes consist of a lipid bilayer, which is a thin polar membrane composed of two sheets of phospholipid molecules. Phospholipids found in mammalian membranes are exclusively zwitterionic or anionic.^1−3^ Although the main part of the lipid carries a net neutral charge, it is well-known that the outer surfaces of the phospholipid bilayer membrane are negatively charged, suggesting that the surface potential can attract and bind positively charged ions, peptide motifs, and proteins.^4−7^ Because to the negative charge on the cell surface, it is difficult for anions to pass through the lipid bilayer.^8−11^ To solve this problem, anion transport systems such as molecular carriers based on amphiphilic cation molecules that transport anions across the lipid membrane have been developed.^12−16^

As building blocks based on aromatic amphiphiles can provide well-organized supramolecular architectures in aqueous environments,^17^ recently reported cationic amphiphiles are mostly composed of hydrophobic segments comprising rigid aromatic rings and hydrophilic segments comprising flexible chains with ammonium- or imidazolium-based groups. Supramolecular complexes of amphiphilic aromatic cations and target anions can be utilized as carriers in amphiphilic membrane environments.^18−25^ For example, González-Mendoza et al. synthesized chiral bis-imidazolium amphiphiles derived from amino acids with N-amide substitution.^13^ Mori et al. designed imidazolinium-based multiblock amphiphiles possessing aromatic rings, oligoethylene glycol chains, and imidazolium moieties.^15^ Casal-Dujat et al. designed imidazolium amphiphiles for anion recognition and transport into the cells. They also studied the delivery of anionic pharmaceuticals such as ibuprofen.^21^ Zheng et al. reported self-assembly of artificial supramolecular channels from protonated amino-triazole amphiphiles to transport anions across the lipid bilayer.^22^

However, most of the reported examples show separation between the aromatic and cationic segments, which are linked by single bonds such as alkyl chains and oligoether chains. Flexible molecular conformations resulting from the rotation of single bonds can limit the scope of well-defined interactions with aromatic anions, leading to the formation of supramolecular nanostructures. In this regard, novel rigid aromatic molecules with close cationic segments are attractive tools for developing systems with enhanced multiple interactions between synthetic amphiphiles and target aromatic anions that exhibit reduced degrees of freedom.

**Figure 1:**
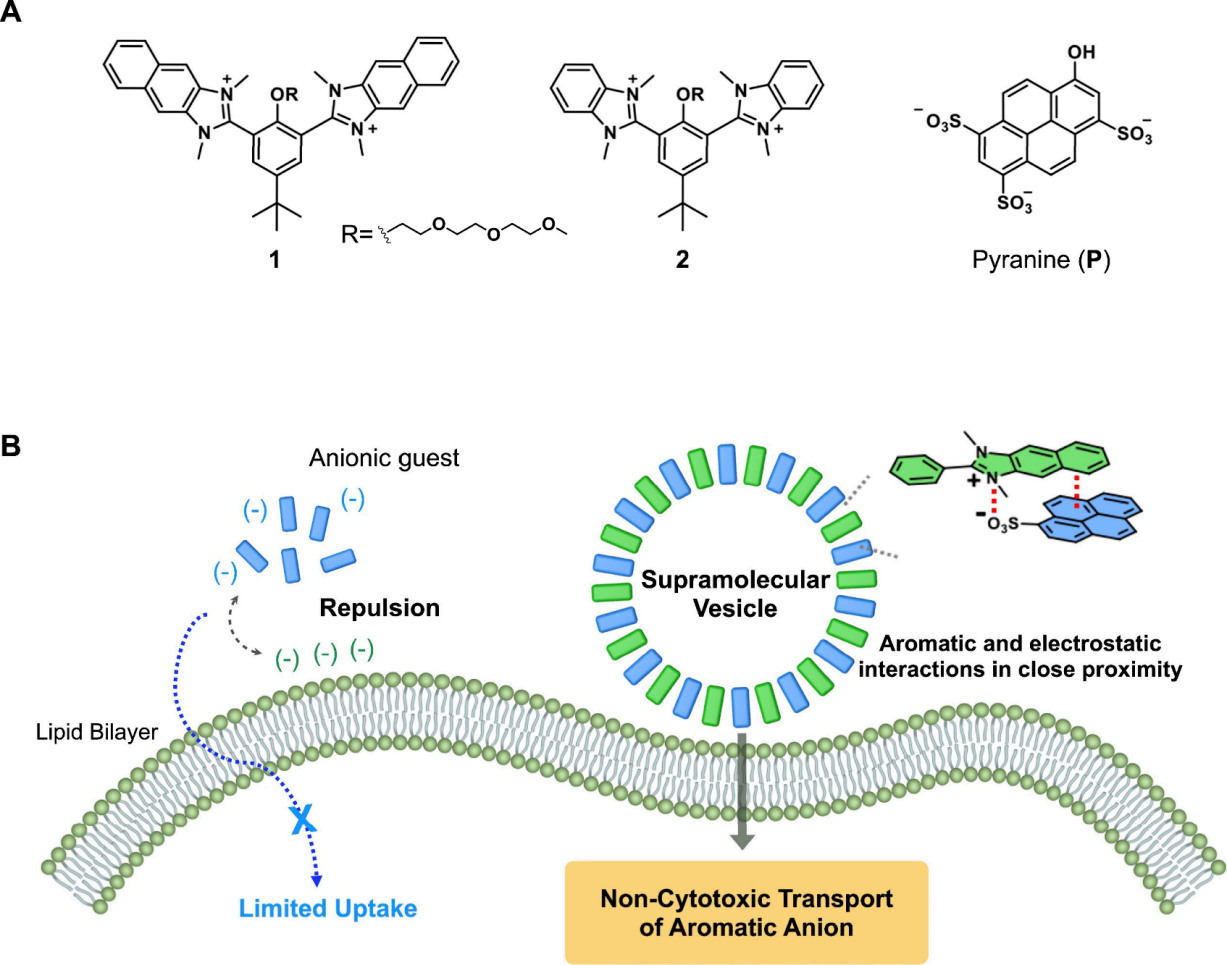
(A) Chemical structures of **1**, **2**, and pyranine. (B) Schematic representation of the anion carriers in supramolecular vesicles via the **1**-pyranine complex.

Herein, we report cationic-fused aromatic amphiphiles composed of aromatic rings and imidazolium groups in close proximity to one another. Molecule **1** was an amphiphilic divalent cationic molecule with two imidazolium segments. The imidazolium groups of **1** neutralized the negatively charged pyranine (8-hydroxypyrene-1,3,6-trisulfonate) through electrostatic interactions, and the naphthalene groups of **1** formed aromatic interactions with the pyrene group of pyranine, producing supramolecular vesicles in an aqueous solution (Figure 1). Furthermore, the vesicles could transport anionic pyranine across the membrane of live cells without significant cytotoxicity.

## Results and Discussion

**Scheme 1:**
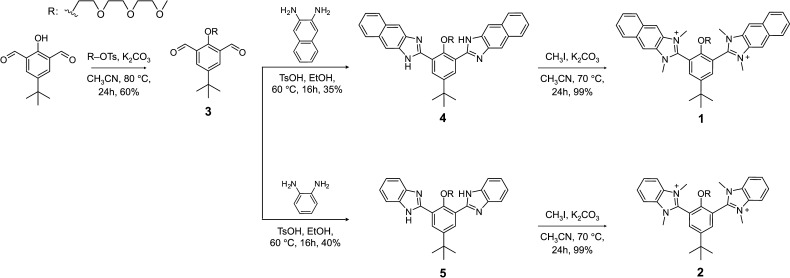
Synthetic Methods for Imidazolium-Fused Amphiphiles **1** and **2**

The synthesis of naphthoimidazolium-based **1** began with the etherification of 4-*tert*-butyl-2,6-diformyphenol (molecule **3**) to incorporate the hydrophilic flexible oligoether chain (Scheme 1 and Scheme S1). A condensation reaction between the aldehydes of **3** and 1,2-diaminonaphthalene was performed to form an imidazole ring on both sides of **3**. The four nitrogen atoms of molecule **4** were methylated with iodomethane to provide the final cationic product **1**. Another cationic amphiphile, **2**, based on benzimidazolium, was also synthesized. The target amphiphilic molecules were characterized by proton and carbon-13 nuclear magnetic resonance spectroscopy (NMR) and high-resolution mass spectroscopy to confirm the structure presented herein (Figure. S1–S14).

**Figure 2:**
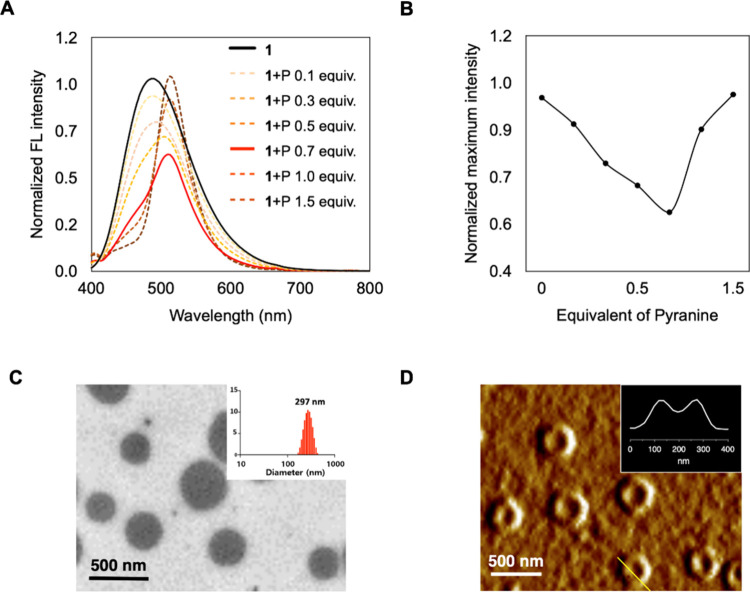
(A) Emission spectra of **1** (106 μM) and different concentrations of pyranine (excitation wavelength 330 nm). (B) Maximum intensity of the emission spectra. (C) TEM image of the complex (**1**; 106 μM and **P**; 74.2 μM) in an aqueous solution. The inset shows the size distribution graph of the DLS measurements. (D) AFM image of the complex (**1**; 106 μM and **P**; 74.2 μM) in an aqueous solution. The inset shows the width profile represented by the yellow line.

The interactions between **1** and pyranine (**P**) were studied by spectroscopy. The absorption spectra of the **1**-pyranine complex exhibited a blue shift at 246–256 nm, which is within the absorption range of **1**, and a red shift from 403 to 405 nm, which is within the absorption range of pyranine. This suggests that intermolecular interactions between **1** and pyranine occur in aqueous environments (Figure S15). When different molar ratios of pyranine were added to **1**, the fluorescence intensity was red-shifted and gradually decreased with pyranine concentration up to 0.7 equiv of pyranine (Figure 2A and 2B). However, when 1.5 equiv of pyranine was added to **1**, an emission spectrum similar to that of free pyranine was clearly observed at 514 nm because of the presence of excess pyranine that could not interact with **1** (Figure S16). This result suggests that the complex formation between **1** and pyranine occurs at a 1:0.7 ratio, corresponding to the net charge ratio of the two molecules. This ratio was used in subsequent experiments.

To investigate the aggregation of the complex, we conducted negative-staining transmission electron microscopy (TEM). The TEM images revealed spherical nanostructures in deionized water with an average size of 297 nm, according to the size distribution data obtained through dynamic light scattering (DLS) measurements (Figure 2C). Atomic force microscopy (AFM) measurements revealed concave vesicles characterized by a depressed central portion and a raised rim due to the topographic properties of the vesicle surface caused by tip-induced vertical artifacts on the vesicles (Figure 2D).^26,27^ In contrast, individual **1** and pyranine do not have characteristic structures in aqueous solutions, unlike the complex (Figure S17). In this experiment, the surface charge was measured using zeta potential analysis. Pyranine and molecule **1** exhibited zeta potentials of −36.1 mV and +10.6 mV, respectively. The complex displayed a surface charge of −17.18 mV, indicating a reduction in negative charge compared to pyranine alone. This suggests that the interaction between **1** and pyranine effectively diminishes the negative charge levels of pyranine (Figure S18).

In the case of **2**, which contains benzene in place of the naphthalene in **1**, the absorption spectrum of pyranine was almost unchanged without a shift near 405 nm after adding 0.7 equiv of pyranine to **2** (Figure S19A and S19B). This result indicates that the interaction between **2** and pyranine was not strong enough for complex formation and that the size and structure of the aromatics at the wing side of the amphiphilic molecule have a significant influence on the interactions with the pyranine anion. Indeed, the TEM images and DLS data of **2** and the **2**-pyranine complex did not show any well-defined structures (Figure S20). Furthermore, molecule **2** exhibited a diverse size distribution in the DLS data because of its insoluble properties.

The interactions between **1** and pyranine in tetrahydrofuran (THF)-water cosolvent were studied because THF–water can mimic the amphiphilic environment of lipid bilayers.^28,29^ Upon the addition of THF, the fluorescence intensity at approximately 490 nm decreased (Figure S21A and S21B). These results indicate that the interactions between the aromatic segments of **1** and pyranine could be strengthened at higher organic solvent ratios where ionic molecules can participate in stronger interactions.^30^ In 100% THF, the absorbance and fluorescence intensity were very weak because of poor ion solubility, which was indicated by the observed precipitation in the solution. Therefore, 90% THF in water was selected for further experiments. The TEM image of the complex in 90% THF revealed many small compact vesicles (approximately 120 nm) that did not exhibit structural collapse, even in an aqueous organic cosolvent (Figure S21C and S21D). Additionally, the zeta potential value under 90% THF conditions was 2.00 mV, indicating a higher positive charge than observed in DW due to a stronger interaction than in DW. DFT calculations (M06-2X, 6-31*) for complex formation in different solvent conditions using the polarizable continuum model (PCM) supported the high binding energy and small distance between **1** and pyranine in THF compared to those in water (Figure S21E). The increase in the binding energy is likely a result of enhanced electrostatic interactions in organic solvents with a low dielectric constant.

**Figure 3:**
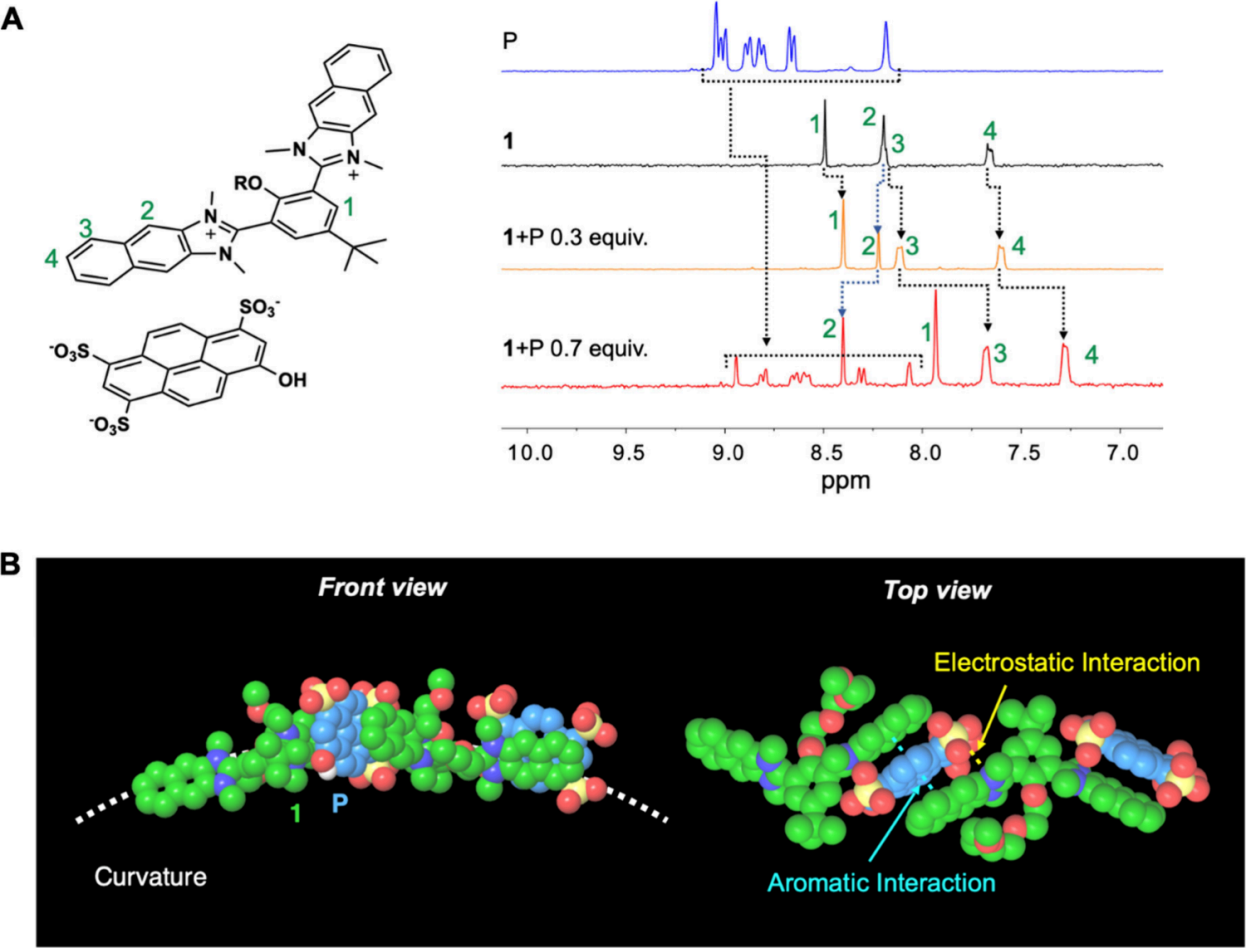
(A) ^1^H NMR spectra of a 74.2 μM pyranine (blue line), 106 μM solution of **1** (black line), 0.3 equiv of **1** (yellow line), and 1.0 equiv complex of molecule **1** with pyranine (red line) in D_2_O. (B) Molecular simulation of the stable complex structure.

NMR analysis provided insights into the intermolecular interactions between molecule **1** and pyranine. The ^1^H NMR spectra of the **1**-pyranine complex (1:0.3 and 1:0.7) in D_2_O displayed notable changes in chemical shifts, reflecting the interaction between the two molecules (Figure 3A). Notably, the aromatic peaks shifted upfield; for example, the H_3_ and H_4_ peaks of **1** shifted from 8.19 to 7.67 ppm and from 7.66 to 7.28 ppm, respectively, and proton peaks of pyranine shifted upfield overall. This is attributed to shielding of the hydrogen nuclei upon aromatic stacking of the two species.^31^ In contrast, H_2_ peak shifted to downfield, from 8.29 to 8.40 ppm because of electrostatic interaction.^32−34^ These observations suggest the presence of both aromatic and electrostatic interactions in the complex of pyranine and molecule **1**. Molecular simulation by density functional theory (DFT) via PBE basis set was conducted to understand the mechanism of the vesicle formations (Figure 3B). The results of the energy minimizations of the complexes, which comprised 2:2 (**1**:pyranine), indicate that adjacent electrostatic and aromatic interactions between the naphthoimidazoliums of **1** and the sulfonate pyrenes of pyranine were the driving forces of the intermolecular interactions (Figure 3B, top view). When the interactions are arranged in two-dimensional directions, the two adjacent aromatic units of **1** and pyranine are packed asymmetrically around the less bulky hydroxy group of pyranine to avoid steric hindrance resulting from the sulfonate groups. This asymmetric packing of aromatic units can induce curvature of the self-assembled layer, resulting in supramolecular vesicles (Figure 3B, front view).

**Figure 4:**
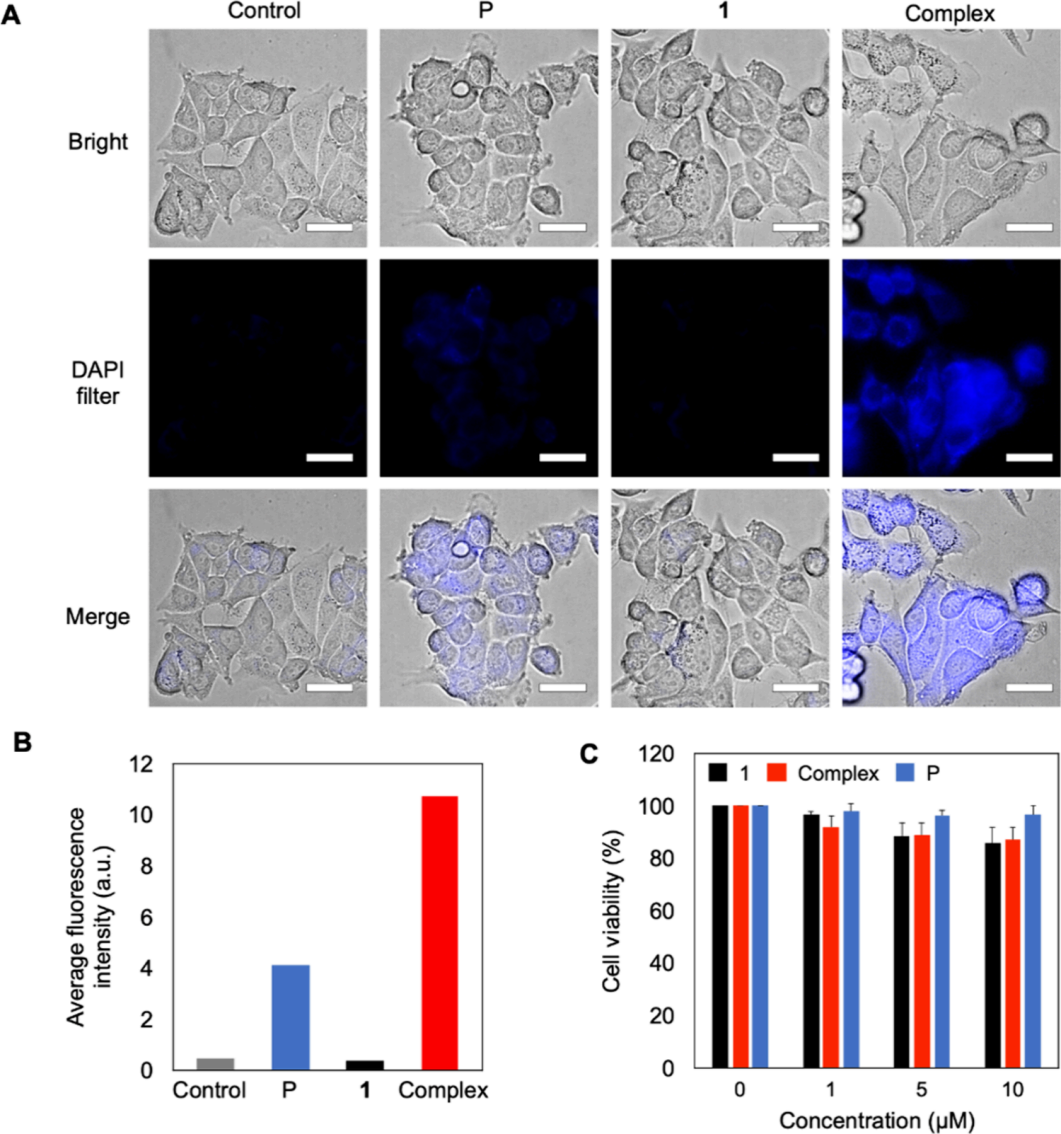
(A) Fluorescence microscopy images of MCF-7 cells treated with 10 μM of **1**, pyranine, and **1**-P complex (excitation: DAPI filter, λ_ex_ = 340–380 nm). Scale bars: 30 μm. (B) Average fluorescence intensity of the microscopy images shown in A. (C) Cell viability of MCF-7 cells, as determined by the MTT cytotoxicity assay.

We used TEM to verify that the vesicle structure of the complex remains intact under cell culture conditions (Figure S22). The observed increase in size measured by DLS compared to that in DW can be attributed to significant aggregation caused by the salt content in the culture media.^35^ The ability of the complex to transport aromatic anionic molecules was tested in live MCF-7 cells. MCF-7 cells were treated with **1**, pyranine, or **1**-pyranine complex and incubated for 4 h. Fluorescence microscopy images showed increased fluorescence in the complex-treated cells, indicating enhanced cellular uptake and successful membrane permeation by the complex (Figure 4A and 4B). This was likely because the **1**-pyranine vesicles could offset the charged species, whereas the anionic pyranine crossed the lipid membrane of the living cells. In addition, the (3-(4,5-dimethylthiazol-2-yl)-2,5-diphenyltetrazolium bromide) tetrazolium (MTT) reduction assay demonstrated low cytotoxicity of **1**-pyranine, indicating that the materials exhibit good biocompatibility (Figure 4C).

## Conclusion

In this study, we employed pyranine, a representative anionic compound, as a model molecule. To address the challenge of anion uptake caused by repulsive charges on the cell membrane, we utilized imidazolium-fused molecule **1**. The interaction between cationic **1** and anionic pyranine resulted in the formation of supramolecular vesicles. These vesicles were formed through multiple interactions between the electrical and aromatic regions of **1** and the anionic aromatic guest. Moreover, supramolecular vesicles have no toxicity and biocompatibility. Considering these results, we expect to form diverse structures based on the length of the aromatic ring and chain type. Building on this, further studies on the effects of various functional groups, aromatic ring structures, solvents, and concentration changes will enhance our understanding of self-assembly morphologies, contributing to the development of more advanced molecular delivery systems. These supramolecular structures, composed of cationic aromatic amphiphiles, have potential applications as effective delivery systems for negatively charged nucleic acids or drugs in the treatment of various diseases.

## Supporting Information Available

Experimental procedure, synthetic methods, ^1^H NMR and ^13^C NMR experiments, high-resolution mass experiments, UV–vis/FL spectroscopy experiments, negatively stained TEM images (PDF)

## ABBREViations

NMR: nuclear magnetic resonance
UV–vis spectroscopy: ultraviolet-vis spectroscopy
TEM: transmission electron microscopy
DLS: dynamic light scattering
AFM: atomic force microscopy
^1^H NMR: proton nuclear magnetic resonance
THF: tetrahydrofuran
MTT assay: 3-(4,5-dimethylthiazol-2-yl)-2,5-diphenyl-tetrazolium bromide assay
FOM: fluorescence optical microscopy
