# Supplementary material for: Aromatic Anion Carrier via Self-Assembly with Imidazolium-Fused Aromatic Amphiphiles
Source: Precis Chem. 2024 Dec 18;3(4):214–20. doi: 10.1021/prechem.4c00074 (PMC12042134; doi:10.1021/prechem.4c00074)
Supplement: Supplementary file 1 — pc4c00074_si_001.pdf [file pc4c00074_si_001.pdf]

Aromatic Anion Carrier via Self-Assembly with Imidazolium-fused Aromatic Amphiphiles

Jung Yeon Park[a], *Dongjun Baek*[a], *Hyunggeun Min*[a], *Bongjun Yeom*[b], *Jeong Sook Ha*[c], *Yongju Kim**[a],[d],[e]

[a] KU-KIST Graduate School of Converging Science and Technology, Korea University, Seoul 02841, Republic of Korea.

Email: yongjukim@korea.ac.kr

[b] Department of Chemical Engineering, Hanyang University, Seoul 24763, Republic of Korea.

[c] Department of Chemical and Biological Engineering, Korea University, Seoul 02841, Republic of Korea.

[d] Department of Integrative Energy Engineering, Korea University, Seoul 02841, Republic of Korea.

[e] Chemical and Biological Integrative Research Center, Korea Institute of Science and Technology, Seoul 02792, Republic of Korea.

KEYWORDS: vesicle, aromatic anion carrier, aromatic amphiphiles, imidazolium, cellular uptake

1. Experimental Procedures

1.1. General methods

All starting materials were obtained from commercial suppliers (TCI, Alfa Aesar, Sigma, etc.) and were used without further purification. Deionized water (DW) was manufactured through ion exchange and filtration; other solvents and organic reagents were purchased by commercial vendors and used without further purification unless otherwise mentioned. All reactions were performed in oven-dried glassware under a dry argon atmosphere. Thin-layer chromatography was performed on precoated glass-backed plates (silica gel 60 F254 0.25 nm), and components were visualized by observation under ultraviolet light (254 and 365 nm) or by treating the plates with iodine, anisaldehyde, $KMnO_4$, phosphomolybdic acid and vanillin followed by heating. The products were purified by flash column chromatography on silica gel (230–400 mesh). ^1H and ^{13}C NMR spectra were obtained from a 400 MHz FT-NMR spectrometer using JNM-ECZ400S/L1. All compounds were subjected to ^1H NMR analysis to confirm ≥98% sample purity. Chemical shifts were reported in ppm relative to the residual solvent peak ($CDCl_3$:^1H, 7.26; ^{13}C, 77.23) or tetramethylsilane (TMS) peak. Multiplicity was indicated as follows: s (singlet), d (doublet), t (triplet), m (multiplet), dd (doublet of doublets). Coupling constants are reported in Hz. Mass spectrometry was performed in ESI (Electrospray Ionization) mass spectrometer (Expression CMS). The UV-vis spectra were measured using the Agilent 8453E UV-visible spectrometer and the fluorescence spectra were obtained from the Hitachi F-7000 fluorescence spectrometer. The size of the aggregates was characterized by dynamic light scattering (DLS) at a fixed angle (90°) using BI-200SM (Brookhaven Instruments Corporation, USA).

1.2. Sample preparation

1, **2** and pyranine were dried in a high vacuum, and a portion of the products was diluted to make a stock solution of 2.13 mM in methanol, respectively. The co-assembled samples were prepared by mixing **1** or **2** and 0.1, 0.3, 0.5, 0.7, 1.0, and 1.5 equivalents of pyranine from stock solution. All organic solvents were evaporated and DW (1 mL) was added to the samples. The samples were sonicated for 60 min and incubated in the dark at room temperature for 16 h.

1.3. Transmission electron microscopy (TEM) experiments

To investigate the self-assembled structures, a drop of the sample solution was placed on a carbon-coated copper grid (carbon type B (15–25 nm) on 200 mesh with Formvar, Ted Pella), and the solvent was allowed to evaporate under ambient conditions. The samples were then stained by depositing a drop of uranyl acetate aqueous solution (0.2–1.0 wt%) onto the surface of the sample-loaded grid. The dried specimens were observed using a Hitachi H-7100 instrument operating at 100 kV.

1.4. Atomic force microscopy (AFM) experiments

The sample film on the mica surface was prepared by evaporation of aqueous **1** (106 μM) solution with 0.7 equivalent of pyranine. Images were obtained in non-contact mode using NX-10 (Park systems) at room temperature. XEI software (Park systems) was used for the analysis of the image.

1.5. Nuclear magnetic resornace (NMR) experiments

The intermolecular interactions between **1** and pyranine were confirmed by ^1H-NMR and NOE. 106 μM of **1**, 74.2 μM of pyranine, and the complex of **1**-pyranine in D_2O were sonicated for 60 min. The sample solutions were measured on a 400 MHz NMR spectrometer.

1.6. Molecular simulations

The complex of two **1** and two pyranine was optimized by DMol3 module based on density functional theory (DFT) using Material Studio 6.0 program with the following parameters; opt energy convergence: 2.0000e-005, opt gradient convergence: 4.0000e-003 A, opt displacement convergence: 5.0000e-003 A, opt iterations: 50, opt max displacement: 0.3000 A, initial hessian: improved,

symmetry: off, spin polarization: restricted, basis: dnd, pseudopotential: none, functional: pbe, aux density: octupole, dftd: TS, integration grid: medium, occupation: thermal 0.0050, cutoff global: 3.6000 angstrom, scf density convergence: 1.0000e-005, scf charge mixing: 2.0000e-001, scf iterations: 50, scf diis: 6 pulay. DFT calculations (M06-2X, 6-31*) for complex formation in water and tetrahydrofuran (THF) solvent conditions were performed through the polarizable continuum model (PCM) using Gaussian 09 software to compare the binding energy and distance between **1** and pyranine.

1.7. Cell cultures

MCF-7 cells (Korean Cell Line Bank, Seoul, Korea) were cultured in Roswell Park Memorial Institute (RPMI) 1640 medium (GibcoTM; ThermoFisher), 10% (*v/v*) fetal bovine serum (FBS, Equitech-Bio), and 1% penicillin/streptomycin (Hyclone, Logan, UT, USA). The culture media was replaced when the cells reached 90–95% confluence. The MCF-7 cells were cultured at 37 ℃ in a humidified incubator with an atmosphere of 5% CO_2.

1.8. FOM experiments

Cells were grown overnight on a confocal dish with 13 mm of a hole. Samples were treated to 15,000 MCF-7 cells seeded per well followed by CO_2 incubation for 4 hours. After 2 times washing with phosphate buffer saline (PBS), cells were observed with a Leica DFC9000 S-CMOS fluorescence microscope camera. The image process was performed with Las X software.

1.9. MTT assay experiments

MCF7 cells were cultured in a 96-well plate (SPL, Korea) at a density of 20 X10^3 cells/well. After 24 hours of incubation, the samples were treated in culture media. After 4 hours of incubation, cells were washed with phosphate buffer saline (PBS), and thiazolyl blue tetrazolium bromide (MTT) reagent (0.5 mg/mL; Sigma-Aldrich, St. Louis, MO, USA) was added to each well with media. Absorbance was measured at 570 nm (Epoch, Biotec, USA) following dissolution in dimethyl sulfoxide (DMSO, Sigma Aldrich).

2. Synthetic Methods

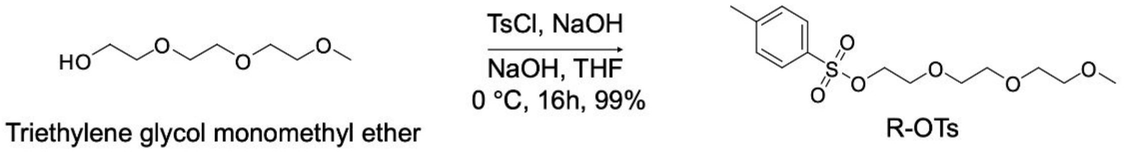

Scheme S1. Synthetic methods for R-OTs.

Compoud **R-OTs**

Triethylene glycol monomethyl ether (5.18 g, 31.547 mmol) was dissolved in tetrahydrofuran (THF). After adding 15 mL of 5 M NaOH (aq) into the THF solution, p-toluenesulfonyl chloride (6.01 g, 31.547 mmol) in tetrahydrofuran was poured slowly into the mixture. The reaction mixture was stirred in the ice bath for 16 hours. After monitoring by TLC, the reaction mixture was extracted with ethyl acetate and dried over $MgSO_4$(s). The filtrate was under reduced pressure by evaporator and vacuum overnight. The white solid is obtained, and the yield is 99% (8.25 g).

^{1}H NMR (400 MHz, $CDCl_3$) δ 7.80–7.78 (m, 2H), 7.36–7.33 (m, 2H), 4.17–4.15 (m, 2H), 3.70–3.51 (m, 12H), 3.37 (s, 3H), 2.45 (s, 3H). ^{13}C NMR (100 MHz, $CDCl_3$) δ 144.90, 133.04, 129.90, 128.03, 71.93, 70.77, 70.54, 69.31, 68.73, 59.04, 21.69. ESI mass: *m/z* calcd. for $C_{14}H_{22}NaO_6S$ $[M+Na]^+$; 341.10, found; 341.0.

Molecule **3**

Compound **R-OTs** (140 mg, 0.4 mmol) and 4-tert-butyl-2 6-di-formyl phenol (125 mg, 0.6 mmol) were dissolved in acetonitrile before adding K_2CO_3 (553 mg, 4.00 mmol). The mixture was refluxed overnight under atmospheric argon. After 24 hours of reaction, the mixture was extracted with ethyl acetate many times and the organic layer was dried over $MgSO_4$(s). The filtrate was condensed by the evaporator and purified by a silica gel flash column using ethyl acetate/hexane (1:2, *v/v*). The yield is 60% (84 mg) as a white solid.

^{1}H NMR (400 MHz, $CDCl_3$) δ 10.46 (d, J = 1.0 Hz, 2H), 8.11 (s, 2H), 4.37–4.35 (m, 2H), 3.91–3.88 (m, 2H), 3.70–3.61 (m, 6H), 3.55–3.52 (m, 2H), 3.34 (s, 3H), 1.37 (s, 9H). ^{13}C NMR (100 MHz, $CDCl_3$) δ 174.96, 147.99, 133.58, 117.28, 115.59, 64.19, 57.68, 56.34, 56.32, 44.58, 20.52, 16.85. ESI mass: *m/z* calcd. for $C_{19}H_{28}NaO_6$ $[M+Na]^+$; 375.18, found; 374.8.

Molecule **4**

Molecule **3** (50 mg, 0.142 mmol) and 2,3-diaminonaphthalene (46 mg, 0.291 mmol) were dissolved in 99% ethanol before adding *p*-toluenesulfonic acid monohydrate (5 mg, 0.029 mmol). The mixture was stirred at 60 ℃ for 16 hours under atmospheric argon. After monitoring of reaction by TLC, the mixture was extracted with ethyl acetate and dried over $MgSO_4$(s). The filtrate was condensed by the evaporator and purified by a silica gel flash column using ethyl acetate/hexane (5:2, *v/v*) as eluent. The yield is 35% (31 mg) as a yellow solid.

^{1}H NMR (400 MHz, $CDCl_3$) δ 12.08 (s, 2H), 8.45 (s, 2H), 8.35 (s, 2H), 8.21 (s, 2H), 8.11–8.07 (m, 4H), 7.53–7.44 (m, 4H), 3.61–3.59 (m, 2H), 3.48–3.43 (m, 4H), 3.38–3.36 (m, 2H), 3.20 (s, 2H), 3.08–3.06(m, 2H), 1.15 (s, 9H). ^{13}C NMR (100 MHz, $CDCl_3$) δ 153.68, 150.42, 148.94, 143.70, 142.91, 131.91, 130.90, 128.31, 126.38, 126.19, 115.59, 111.56, 71.58, 70.47, 70.07, 58.31, 30.84, 29.67. ESI mass: *m/z* calcd. for $C_{39}H_{41}N_4O_4$ $[M+H]^+$; 629.30, found; 629.5.

Molecule **5**

Molecule **3** (20 mg, 0.057 mmol) and 1,2-phenylenediamine (13 mg, 0.120 mmol) were dissolved in 99% ethanol before adding p-toluenesulfonic acid monohydrate (2 mg, 0.012 mmol). The mixture was stirred at 60 ℃ for 16 hours under atmospheric argon. After monitoring of reaction by TLC, the mixture was extracted with ethyl acetate and dried over $MgSO_4$(s). The filtrate was condensed by the evaporator and purified by a silica gel flash column using ethyl acetate as eluent. The yield is 40% (12 mg) as an orange color solid.

^{1}H NMR (400 MHz, $CDCl_3$) δ 11.99 (s, 2H), 8.21 (s, 2H), 7.94–7.93 (d, *J* = 7.5 Hz, 2H), 7.75–7.73 (d, *J* = 7.5 Hz, 2H), 7.38–7.35 (dd, *J* = 8.4, 2.7 Hz, 4H), 3.62–3.42 (m, 10H), 3.27 (s, 3H), 3.19–3.16 (m, 2H), 1.15 (s, 9H). ^{13}C NMR (100 MHz, $CDCl_3$) δ 151.94, 149.70, 147.23, 143.20, 135.03, 129.61, 119.13, 111.45, 72.94, 71.78, 70.46, 70.18, 69.90, 68.91, 60.39, 58.94, 34.38, 30.77. ESI mass: *m/z* calcd. for $C_{31}H_{37}N_4O_4$ $[M+H]^+$; 529.27, found; 529.4.

Molecule **1**

Molecule **4** (15 mg, 0.024 mmol) was dissolved in acetonitrile, and K_2CO_3 (10 mg, 0.072 mmol) and iodomethane (40 µL, 0.642 mmol) were added to the solution. The mixture was stirred at 70 ℃ for 1 day under atmospheric argon. After monitoring by mass spectrometry and TLC, the mixture was filtrated and dried under reduced pressure by an evaporator and vacuum. The yield is 99% (16 mg) as a yellowish solid.

^{1}H-NMR (400 MHz, $CDCl_3$:CD_3OD 1:1, *v/v*) δ 8.55 (s, 4H), 8.34 (s, 2H), 8.25–23 (dd, *J* = 6.4, 3.3 Hz, 4H), 7.75–7.73 (dd, *J* = 6.5, 3.2 Hz, 4H), 4.22 (s, 12H), 3.56–3.54 (m, 2H), 3.42–3.39 (m, 4H), 3.27–3.25 (m, 2H), 3.20–3.18 (m, 4H), 1.54 (s, 9H). ^{13}C NMR (100 MHz, $CDCl_3$) δ 151.56, 150.73, 136.34, 132.93, 131.80, 129.08, 127.87, 126.59, 124.22, 116.95, 114.74, 111.63, 72.30, 70.52, 70.41, 70.08, 69.56, 59.04, 42.23, 36.34, 34.48, 31.52. ESI mass: *m/z* calcd. for $C_{43}H_{50}N_4O_4$ $[M]^{2+}$; 343.19, found; 343.1492.

Molecule **2**

Molecule **5** (20 mg, 0.038 mmol) was dissolved in acetonitrile, and K_2CO_3 (12 mg, 0.087 mmol) and iodomethane (50 µL, 0.803 mmol) were added to the solution. The mixture was stirred at 70 ℃ for 1 day under atmospheric argon. After monitoring by mass spectrometry and TLC, the mixture was filtrated and dried under reduced pressure by an evaporator and vacuum. The yield is 99% (22 mg) as a yellow solid.

^{1}H NMR (400 MHz, $CDCl_3$) δ 8.02 (s, 2H), 7.86–7.84 (dd, *J* = 6.3, 3.2, Hz, 4H), 7.76–7.74 (dd, *J* = 6.3, 3.1, 4H) 4.06 (s, 12H), 3.52–3.49 (m, 2H), 3.46–3.45 (m, 4H), 3.36 (s, 3H), 3.13–3.11 (m, 2H), 3.05 – 3.02 (m, 2H), 2.88–2.86 (m, 2H), 2.00 (s, 9H). ^{13}C NMR (100 MHz, $CDCl_3$) δ 155.29, 150.95, 146.31, 134.89, 132.03, 127.98, 116.07, 113.09, 71.83, 70.02, 69.73, 69.56, 68.49, 58.72, 35.40, 33.05, 30.68. ESI mass: *m/z* calcd. for $C_{35}H_{46}N_4O_4$ $[M]^{2+}$; 293.18, found; 293.1783

3. Supplementary Figures

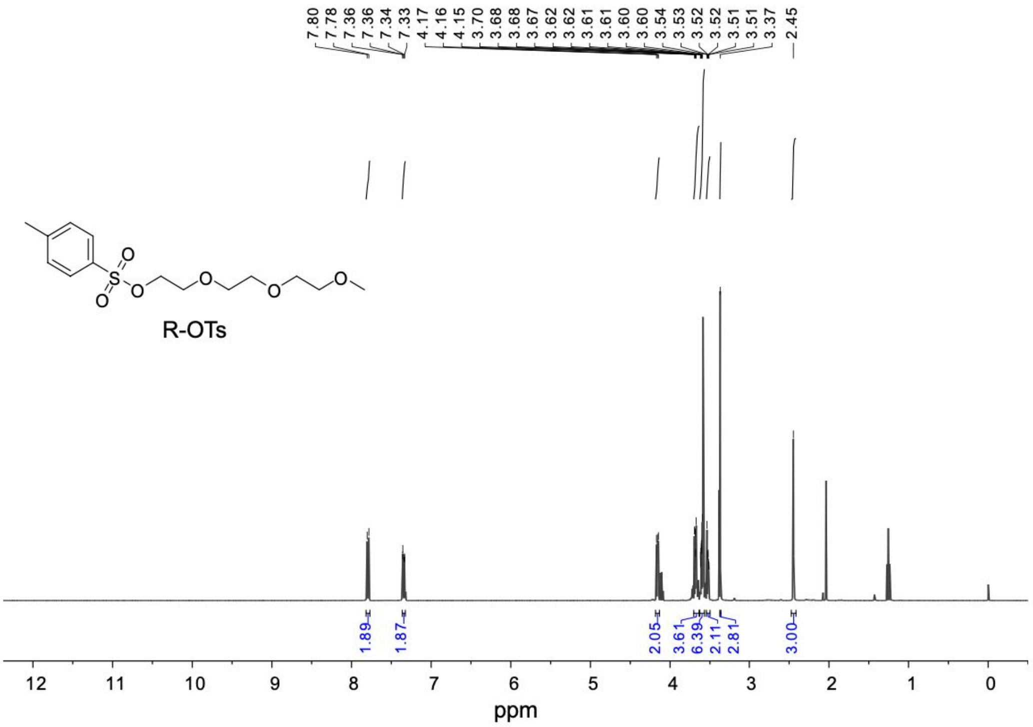

Figure S1. ^{1}H-NMR spectrum of R-OTs in $CDCl_3$.

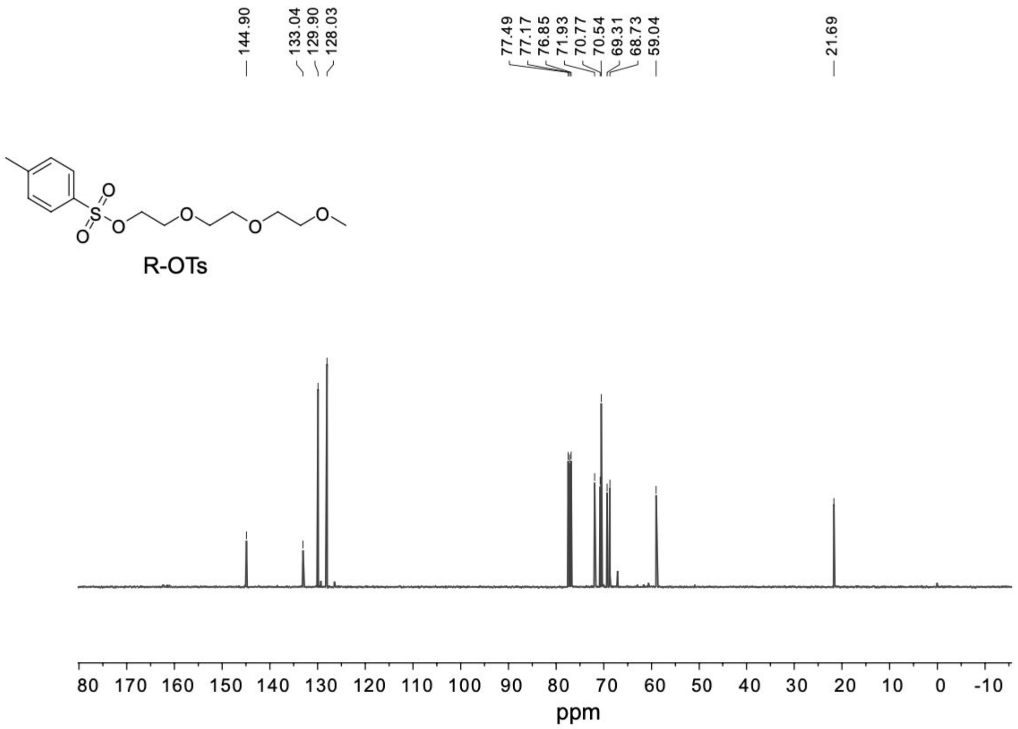

Figure S2. ^{13}C-NMR spectrum of R-OTs in $CDCl_3$.

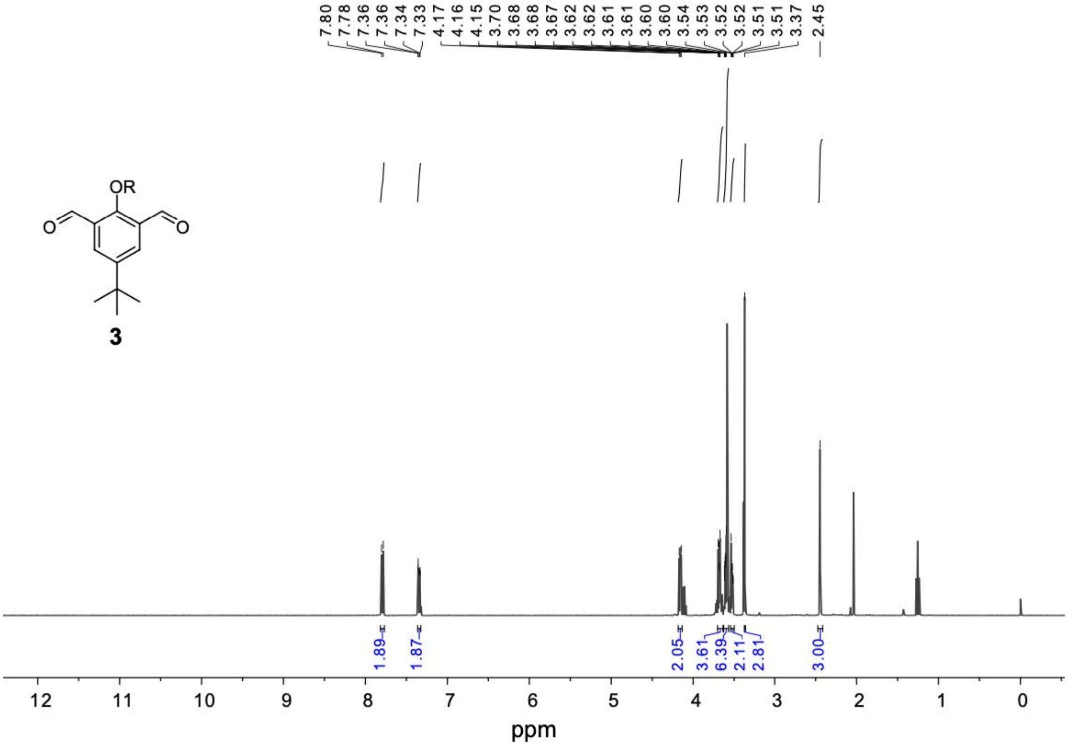

Figure S3. ^{1}H-NMR spectrum of **3** in $CDCl_3$.

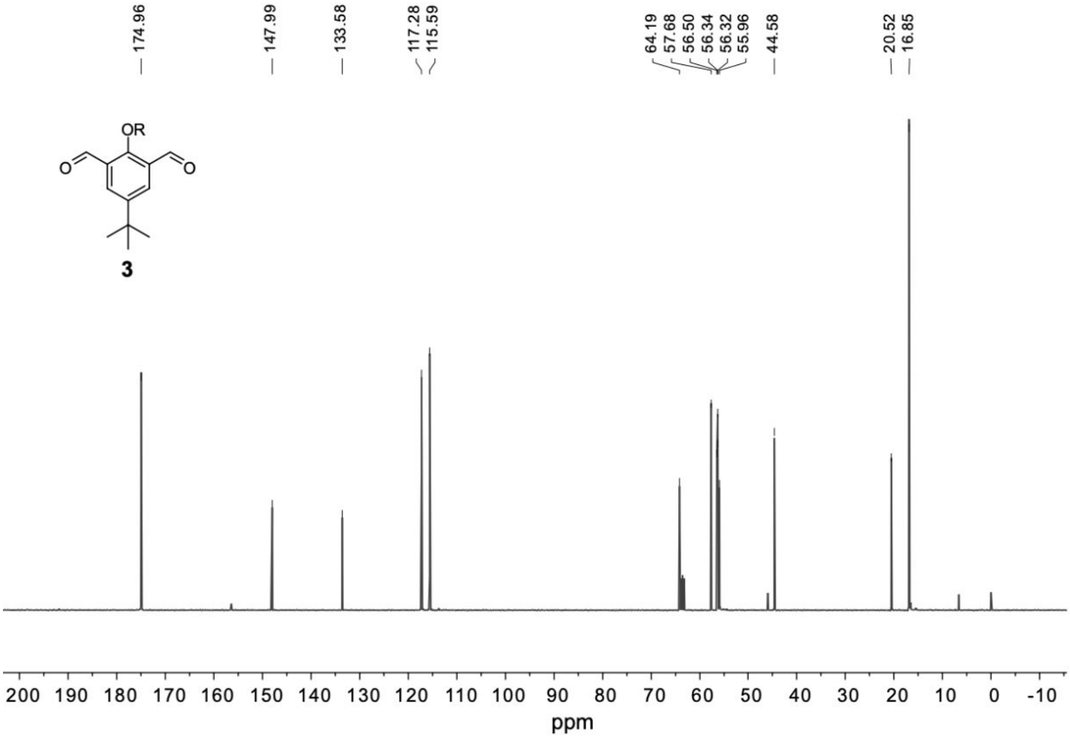

Figure S4. ^{13}C-NMR spectrum of **3** in $CDCl_3$.

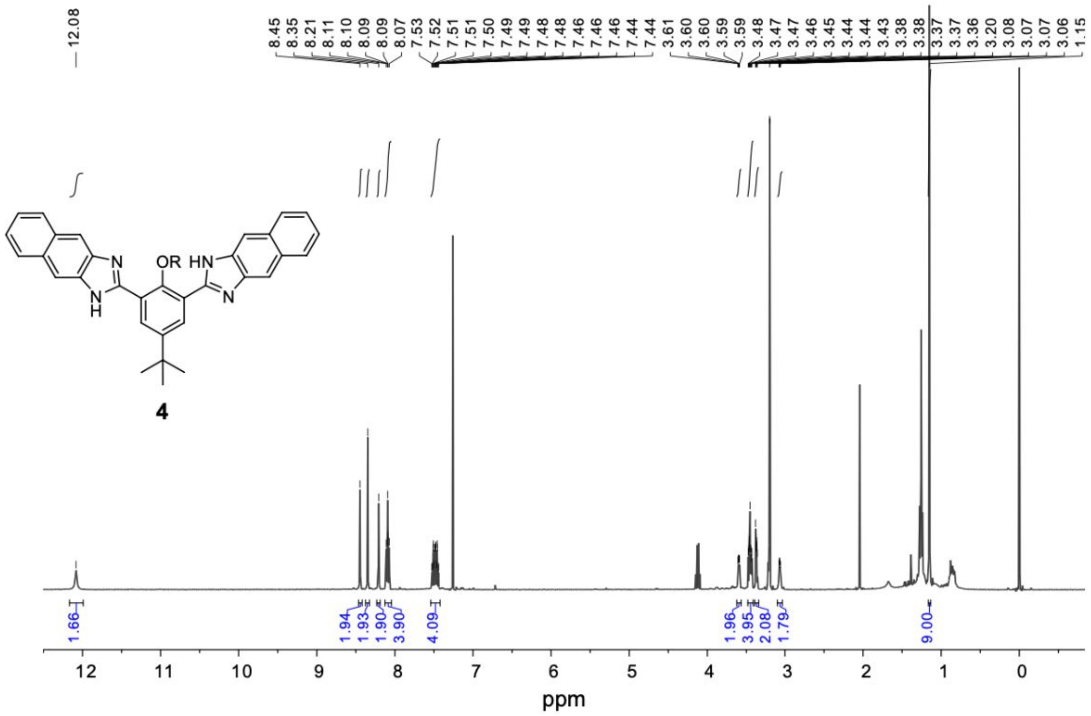

Figure S5. ^{1}H-NMR spectrum of **4** in $CDCl_3$.

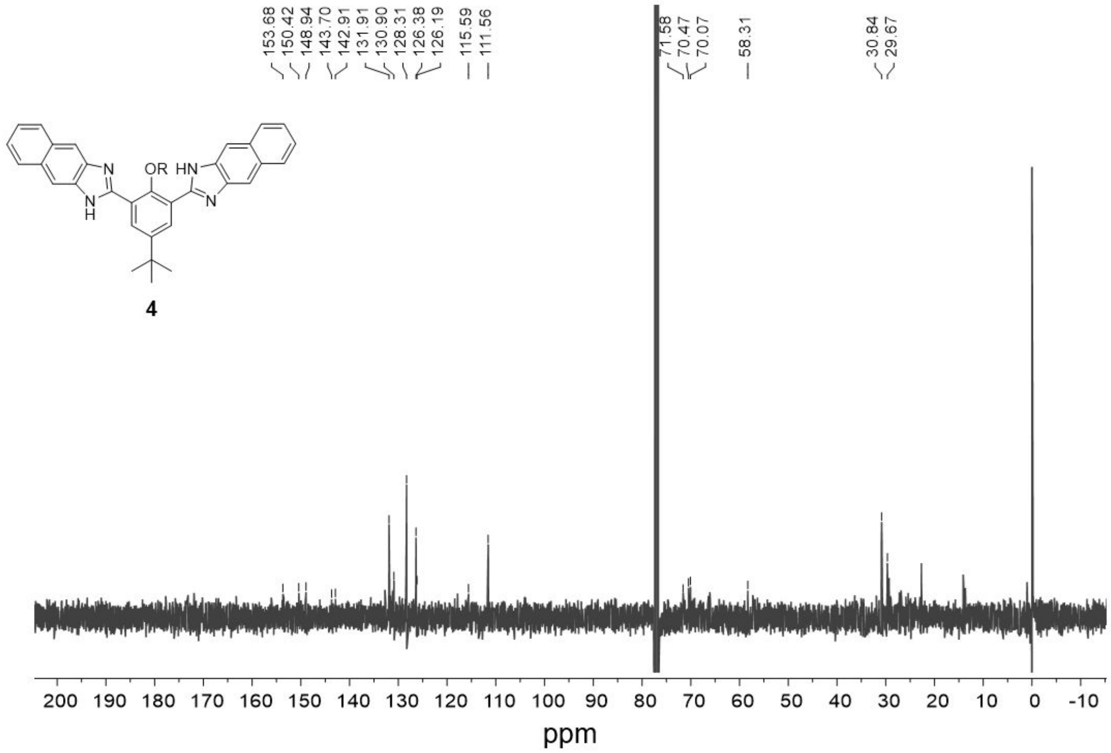

Figure S6. ^{13}C-NMR spectrum of **4** in $CDCl_3$.

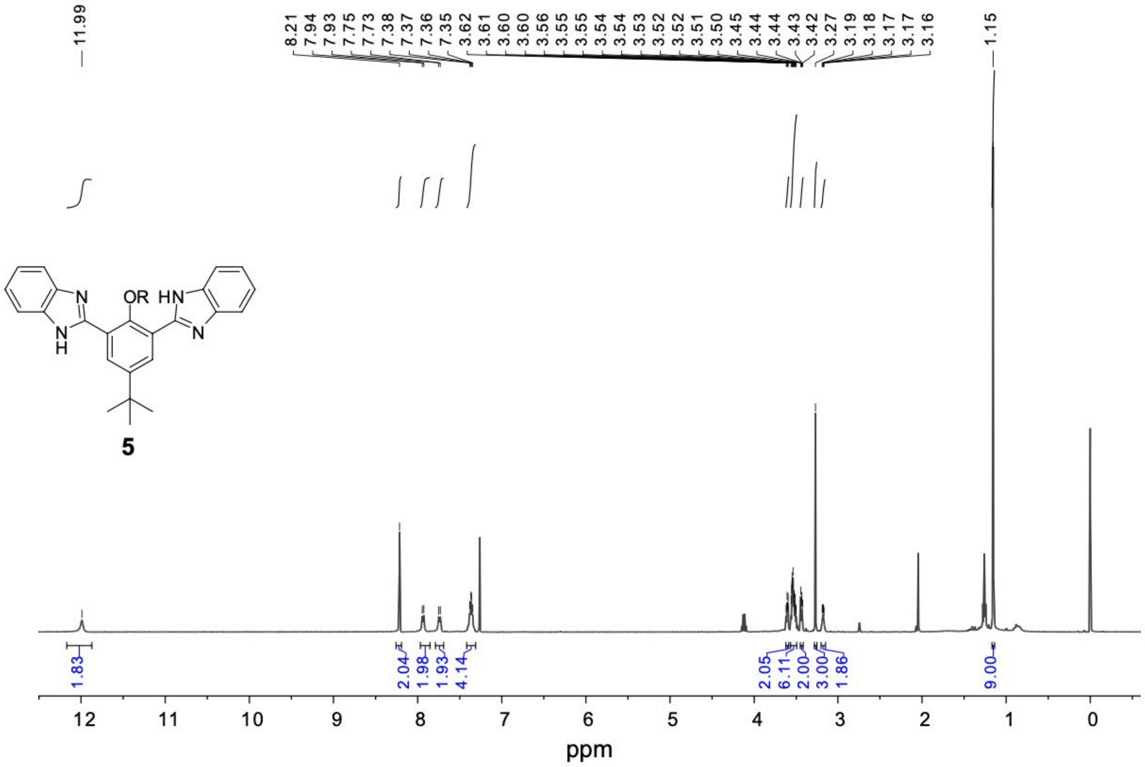

Figure S7. ^{1}H-NMR spectrum of **5** in $CDCl_3$.

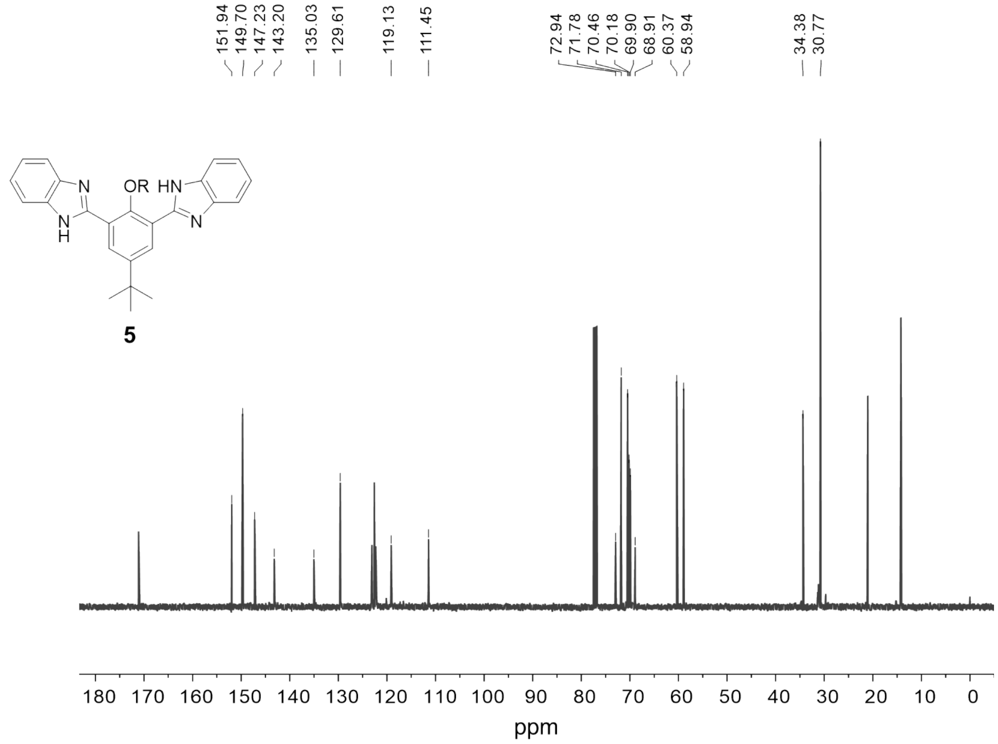

Figure S8. ^{13}C-NMR spectrum of **5** in $CDCl_3$.

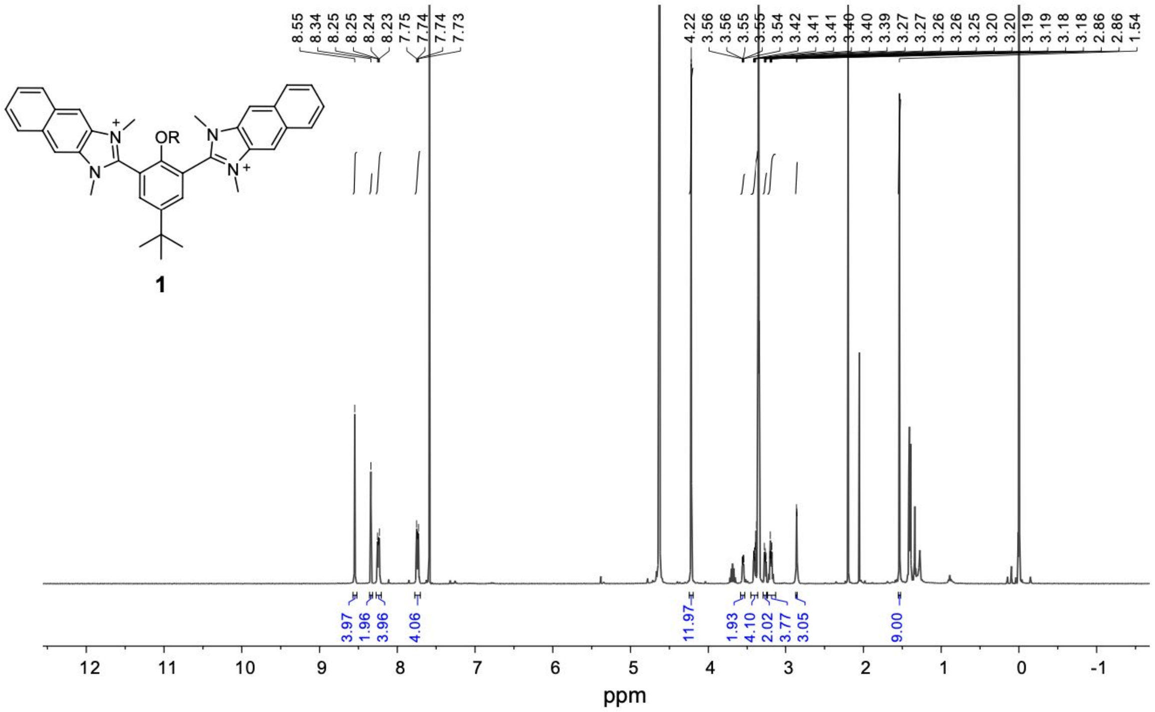

Figure S9. ^{1}H-NMR spectrum of **1** in $CDCl_3$.

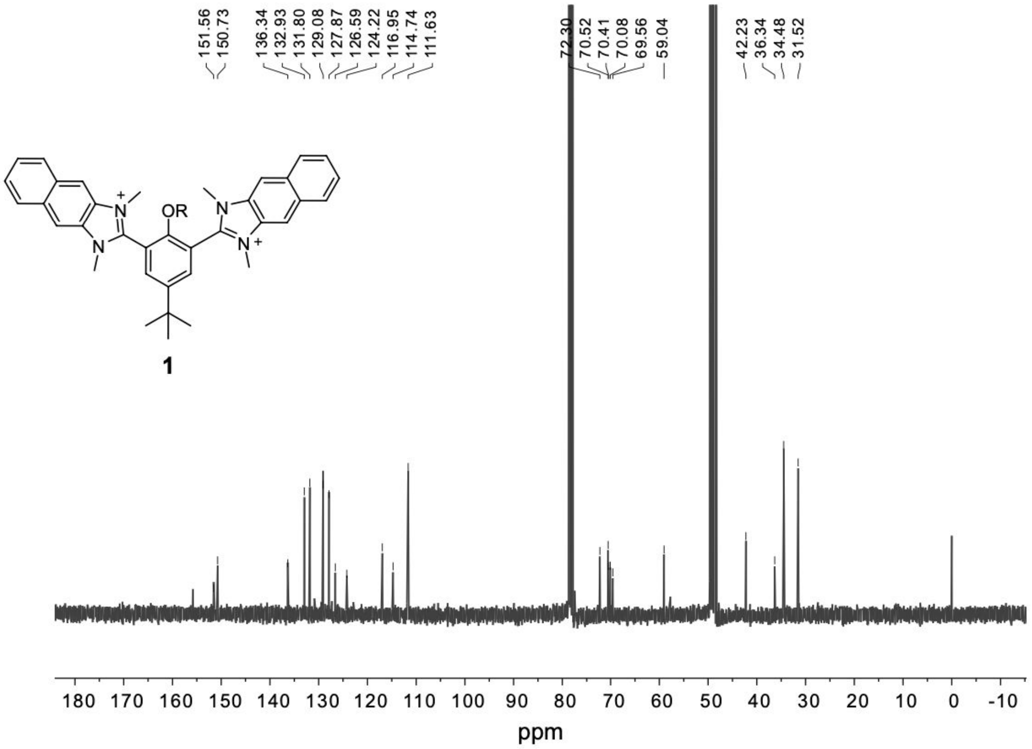

Figure S10. ^{13}C-NMR spectrum of **1** in $CDCl_3$

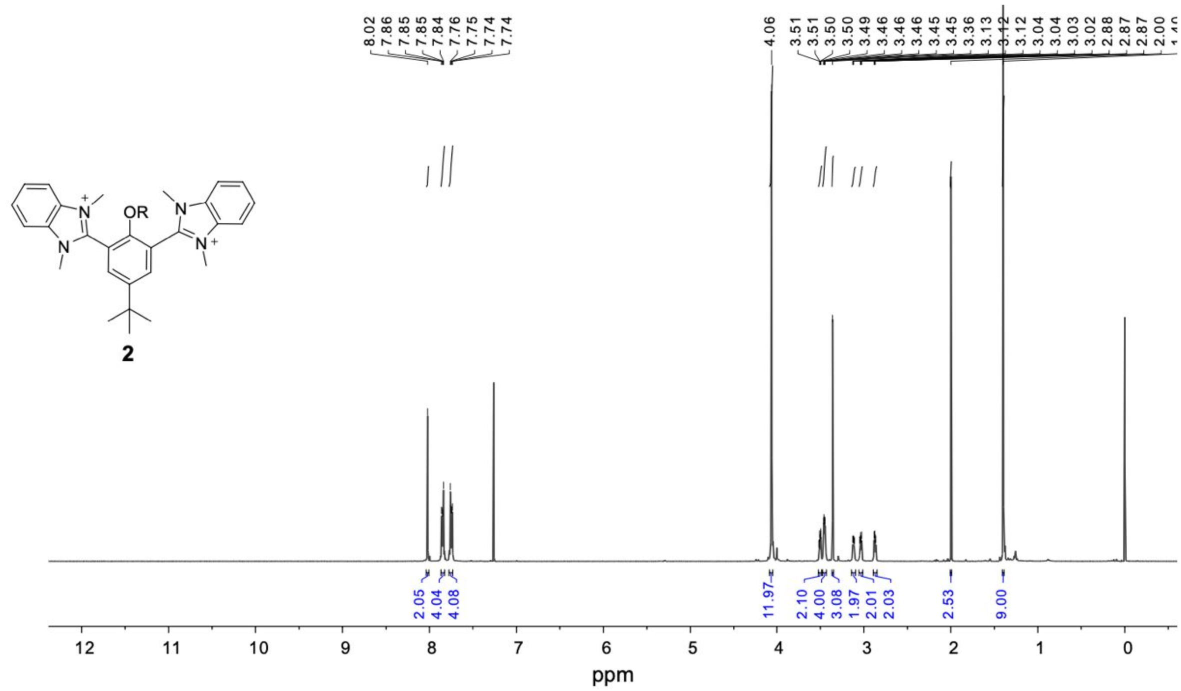

Figure S11. ^{1}H-NMR spectrum of **2** in $CDCl_3$.

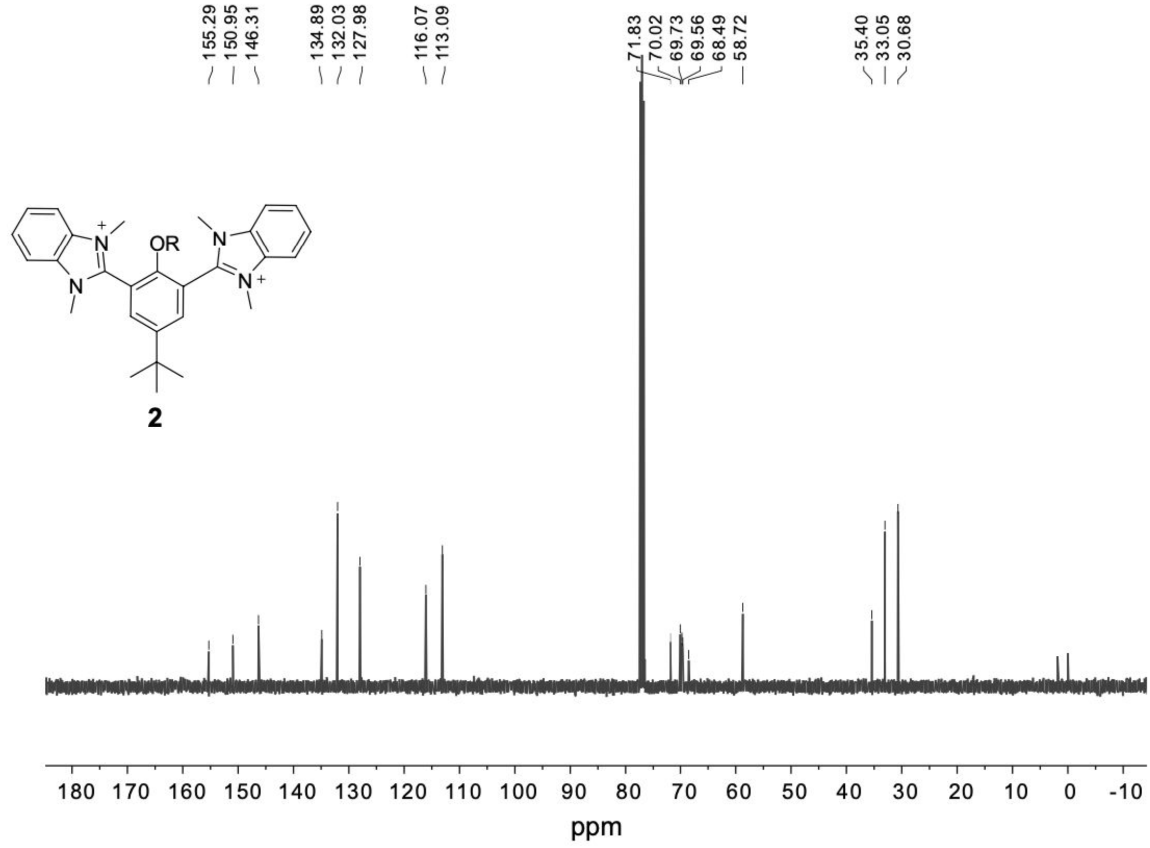

Figure S12. ^{13}C-NMR spectrum of **2** in $CDCl_3$

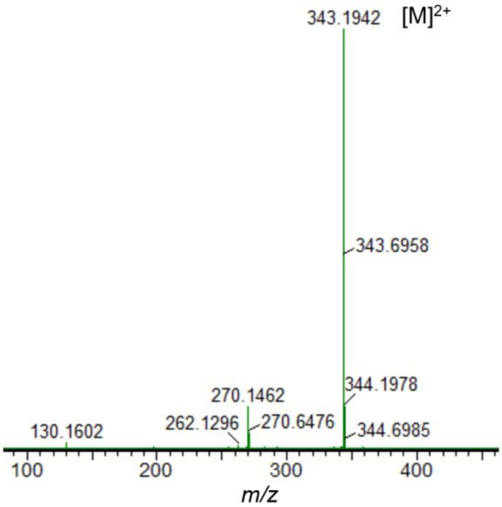

Figure S13. High-resolution mass spectrum of **1**.

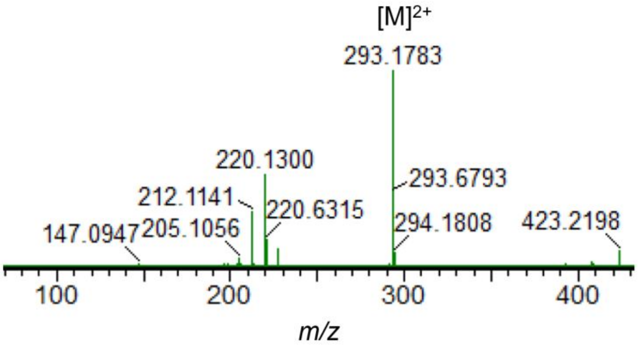

Figure S14. High-resolution mass spectrum of **2**.

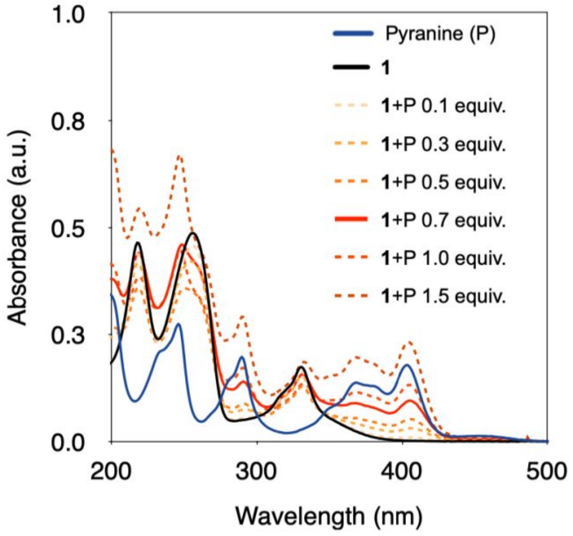

Figure S15. Absorption spectra of **1** (106 μM) with different equivalents of pyranine in aqueous solution.

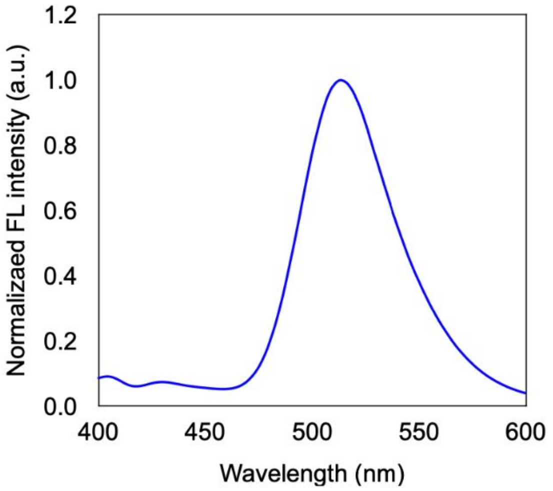

Figure S16. Emission spectrum of pyranine (74.2 μM) in aqueous solution at excitation wavelength 330 nm.

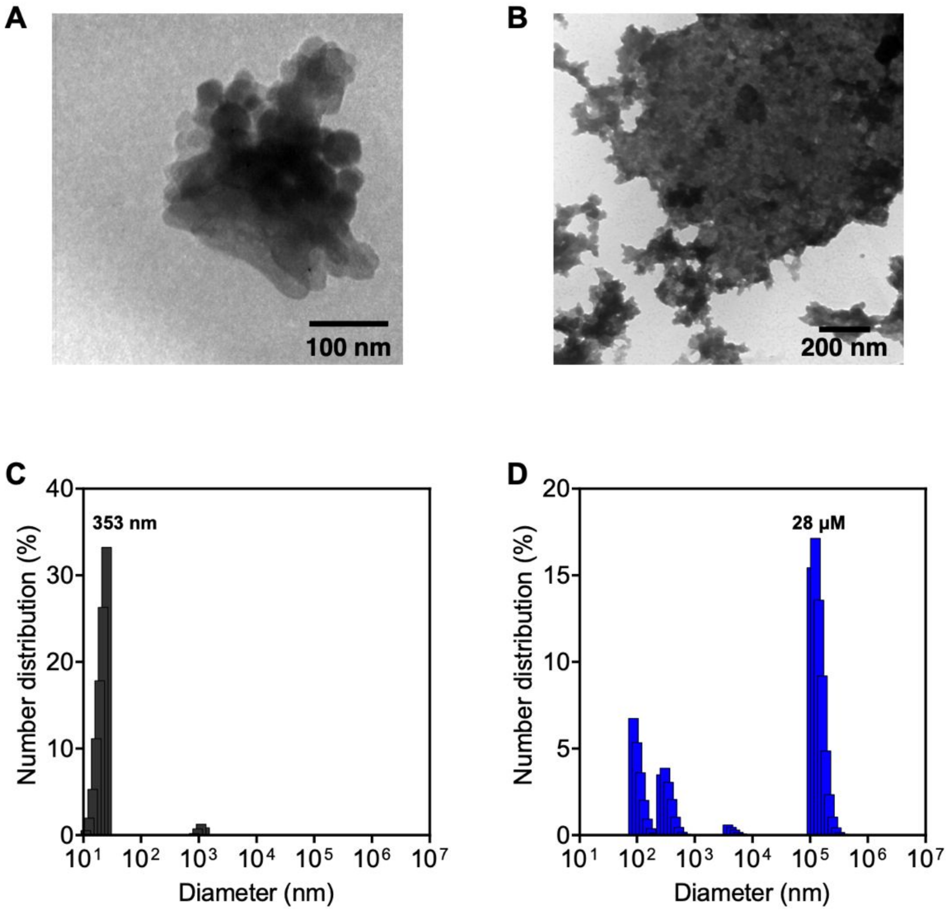

Figure S17. Negatively-stained TEM images of A) **1** (106 µM) and B) pyranine (74.2 µM) in aqueous solution. Size distribution graph of the DLS measurements of C) **1** and D) pyranine.

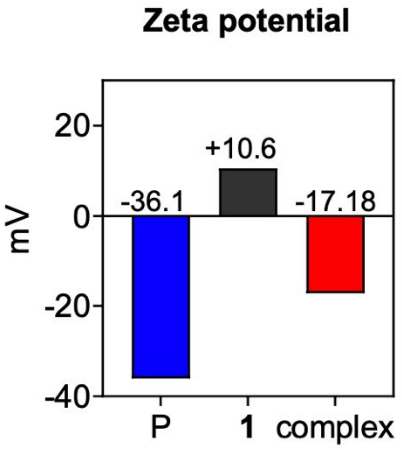

Figure S18. Zeta potential of pyranine (74.2 μM), **1** (106 μM), and complex in aqueous solution.

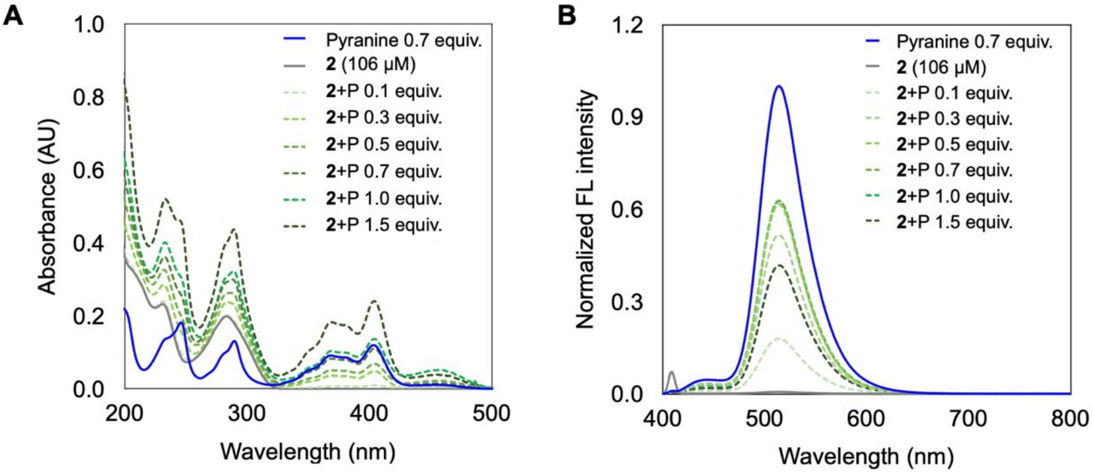

Figure S19. A) Absorption spectra of **2** (106 μM) with different equivalents of pyranine in aqueous solution. B) Emission spectra of pyranine and **2**-pyranine complex in aqueous solution.

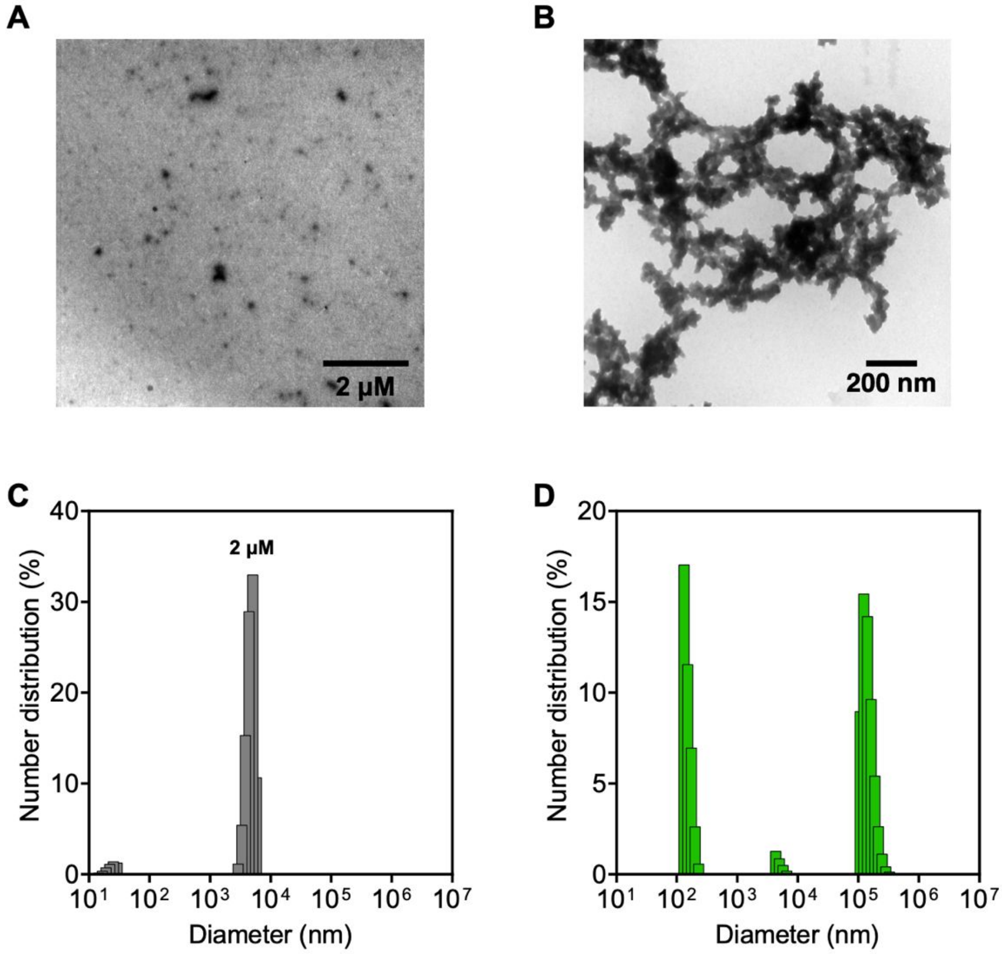

Figure S20. Negatively-stained TEM images of A) **2** (106 μM) and B) **2-**pyranine complex in DW. Size distribution graph of the DLS measurements of C) **2** and D) **2-**pyranine complex.

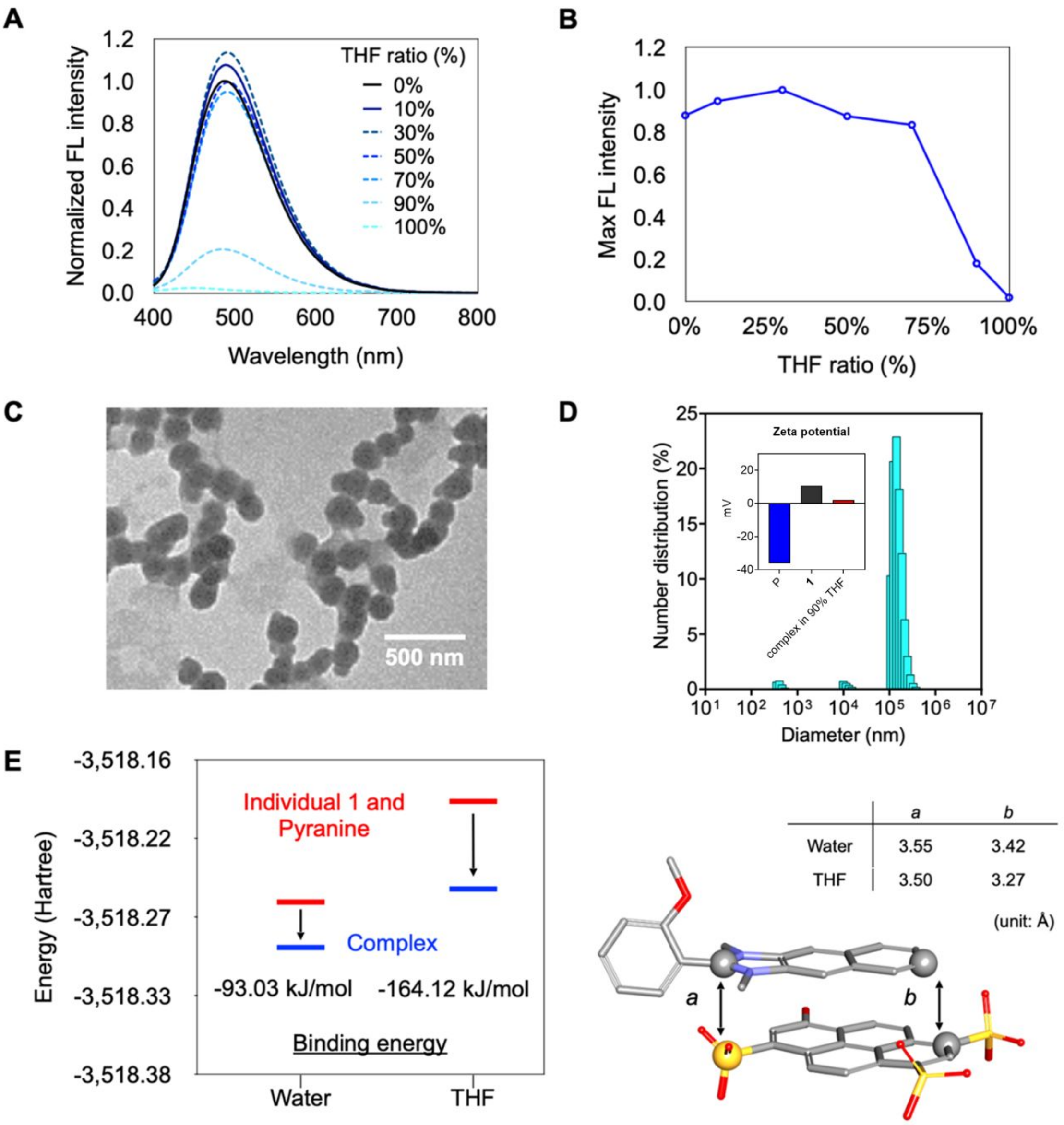

Figure S21. A) Emission spectra of the complex (106 μM) under different THF-water conditions. Excitation wavelength: 330 nm. B) Max FL intensity under different THF-water conditions. C) Negatively-stained TEM image of the complex (106 μM) in 90% THF. D) Size distribution graph of the DLS measurements of the complex (106 μM) in 90% THF. The inset shows the zeta potential of the complex in 90% THF. E) Energy calculations of binding energy and distance between **1** and pyranine, respectively water or THF conditions.

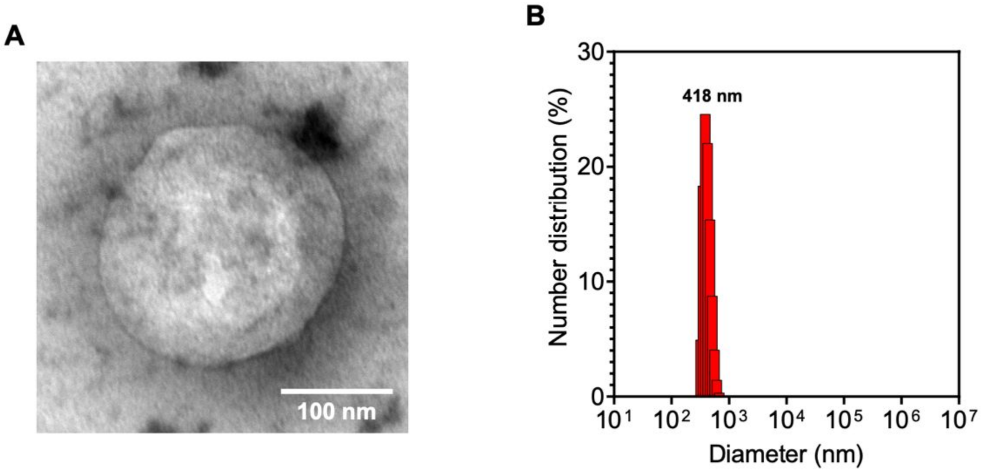

Figure S22. A) Negatively-stained TEM of complex in RPMI media. B) Size distribution graph of the complex in RPMI media.
